# Supplementary figures and images for: Unified platform for genetic and serological detection of COVID-19 with single-molecule technology
Source: PLoS One. 2021 Jul 26;16(7):e0255096. doi: 10.1371/journal.pone.0255096 (PMC8312974; doi:10.1371/journal.pone.0255096)

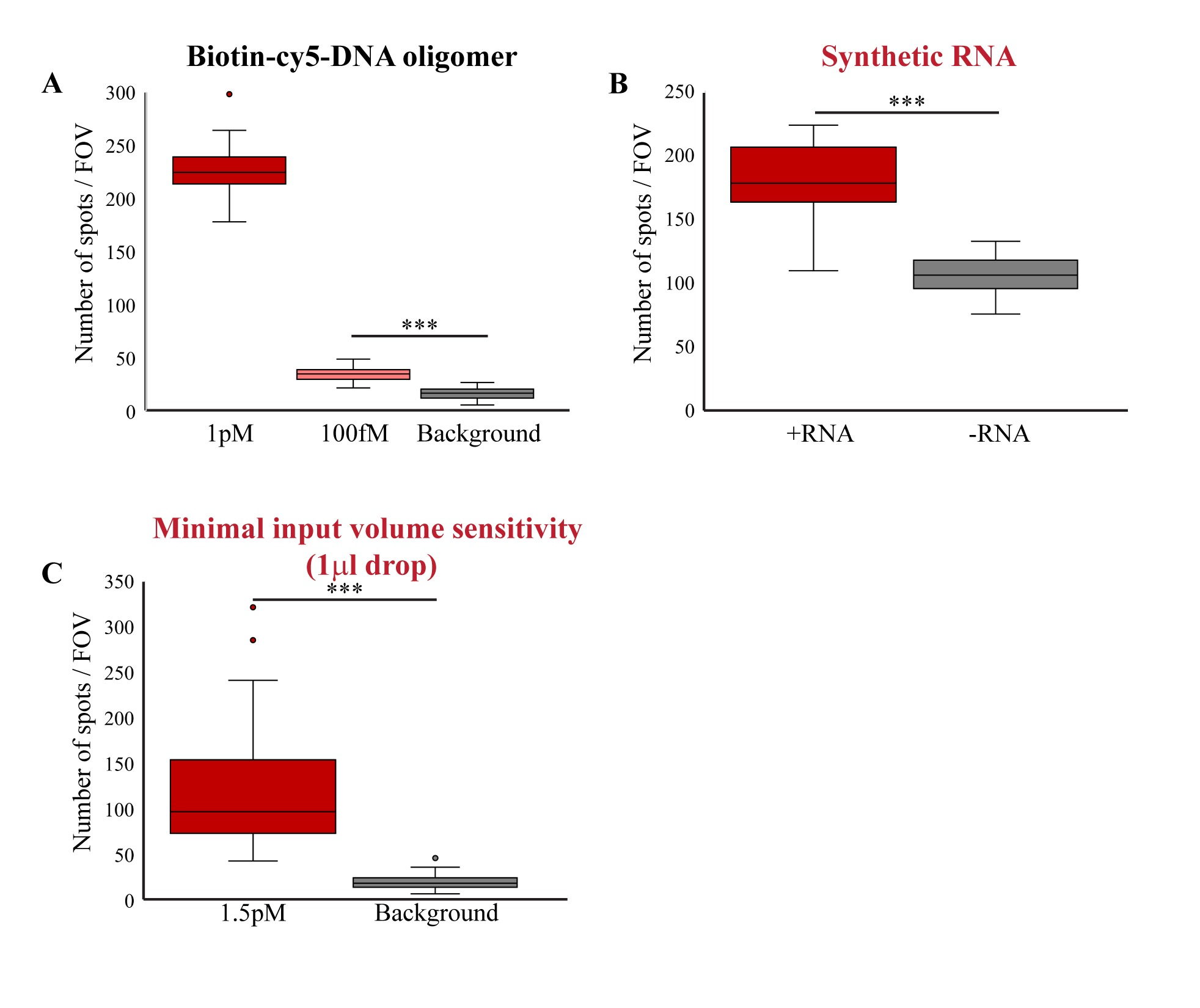

Supplement: S1 Fig — (A) Single-molecule detection of Cy5-DNA probes. Biotin and Cy5 labeled DNA probes at the indicated concentrations were added to a streptavidin-coated surface and imaged by TIRF. *** p-value <0.001. (B) SARS-CoV-2 synthetic RNA (Twist Bioscience) was incubated with capture and detection probes and analyzed as in Fig 1B. *** p-value <0.001. (C) COVID-19 synthetic DNA was incubated with capture and detection probes. A 1μl drop of the hybridized sample was immobilized on a streptavidin-coated surface and imaged. For background assessment, capture and detection probes were incubated with no synthetic DNA. *** p-value <0.001. (TIF) [file pone.0255096.s001.tif]

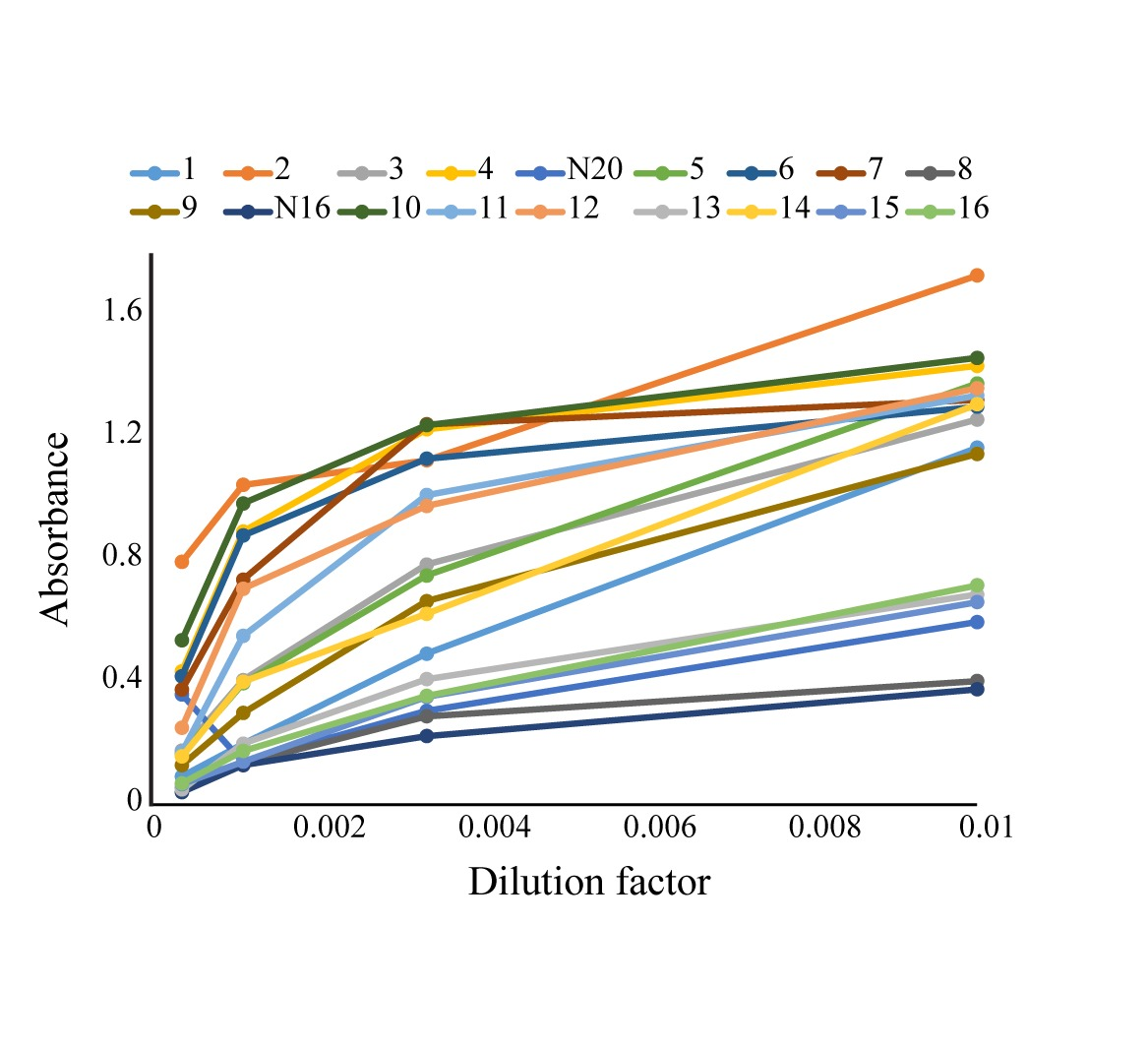

Supplement: S2 Fig — (TIF) [file pone.0255096.s002.tif]
